# Reference genes for quantitative real-time PCR normalization of *Cenostigma pyramidale* roots under salt stress and mycorrhizal association

**DOI:** 10.1590/1678-4685-GMB-2020-0424

**Published:** 2021-05-31

**Authors:** Gabriella Frosi, José Ribamar Costa Ferreira-Neto, João Pacífico Bezerra-Neto, Laís Luana de Lima, David Anderson de Lima Morais, Valesca Pandolfi, Ederson Akio Kido, Leonor Costa Maia, Mauro Guida Santos, Ana Maria Benko-Iseppon

**Affiliations:** 1 Universidade Federal de Pernambuco, Departamento de Botânica, Recife, PE, Brazil.; 2 Université de Sherbrooke, Départament de Biologie, Faculté des Sciences, Sherbrooke, QC, Canada.; 3 Universidade Federal de Pernambuco, Departamento de Genética, Recife, PE, Brazil.; 4 Université de Sherbrooke, Faculté des Sciences, Centre de Calcul Scientifique, Sherbrooke, QC, Canada.; 5 Universidade Federal de Pernambuco, Departamento de Micologia, Recife, PE, Brazil.

**Keywords:** Arbuscular mycorrhizal fungi, Catingueira, RT-qPCR, RNA-Seq, salinity

## Abstract

*Cenostigma pyramidale* is a native legume of the Brazilian semiarid region which performs symbiotic association with arbuscular mycorrhizal fungi (AMF), being an excellent model for studying genes associated with tolerance against abiotic and biotic stresses. In RT-qPCR approach, the use of reference genes is mandatory to avoid incorrect interpretation of the relative expression. This study evaluated the stability of ten candidate reference genes (CRGs) from *C*. *pyramidale* root tissues under salt stress (three collection times) and associated with AMF (three different times of salinity). The *de novo* transcriptome was obtained via RNA-Seq sequencing. Three algorithms were used to calculate the stability of CRGs under different conditions: (i) global (Salt, Salt+AMF, AMF and Control, and collection times), (ii) only non-inoculated plants, and (iii) AMF (only inoculated plants). *HAG2*, *SAC1*, *aRP3* were the most stable CRGs for global and AMF assays, whereas *HAG2*, *SAC1*, *RHS1* were the best for salt stress assay. This CRGs were used to validate the relative expression of two up-regulated transcripts in Salt2h (*RAP2-3* and *PIN8*). Our study provides the first set of reference genes for *C*. *pyramidale* under salinity and AMF, supporting future researches on gene expression with this species.

## Introduction

In the Brazilian semiarid region, there is a seasonally dry tropical forest called Caatinga. Due to the restricted and irregular rainfall, Caatinga plants are often exposed to abiotic stress, such as drought and salinity (De Albuquerque *et al*., 2012; Santos *et al*., 2014)⁠. *Cenostigma pyramidale* (Tul.) L.P. Queiroz (previously called *Poincianella pyramidalis*), popularly known as ‘Catingueira’, is a woody legume tree and figure as one of the most representative species in the Caatinga drylands. This species has multiple uses, such as logging and medicinal applications (Alviano *et al*., 2008; Vilela *et al*., 2020). In addition, other aspects of *C*. *pyramidale* have been studied, such as genetic diversity (Santos *et al*., 2012) and phenotypic plasticity on ecological succession gradients in the semiarid area (Falcão *et al*., 2015)⁠. Recent studies on this species revealed an association of arbuscular mycorrhizal fungi (AMFs) with most efficient physiological and biochemical responses under drought (Frosi *et al*., 2016) ⁠and salinity (Frosi *et al*., 2018).

Due to such promising performance for *C*. *pyramidale u*nder the mentioned abiotic stresses, besides the contribution of AMFs, this plant species became a noteworthy target for molecular studies. In this way, our group performed RNA-Seq of *C*. *pyramidale* root tissues submitted to salinity and associated with AMFs (Frosi *et al*., 2021). Thus, numerous transcripts possibly involved with the tolerance response and higher physiological performance were modulated and require validation.

Quantitative reverse transcription PCR (RT-qPCR) is one of the most robust and reliable techniques for validating high-throughput gene expression studies due to its sensibility and specificity (Sinha *et al*., 2015)⁠. Variables such as the integrity and purity of RNA, the efficacy of reagents used in RNA extraction, sample quantification, and reverse transcription (Bustin *et al*., 2009)⁠ may lead to quantitative and qualitative differences between the evaluated samples, demanding the selection of appropriate reference genes (RGs) for normalization of gene expression (Gutierrez *et al*., 2008; Wang *et al*., 2017)⁠. The critical point to select RGs is to verify if their expression is constitutive in the studied tissues under all evaluated conditions. The proper use of RGs ensures that the observed variation in the relative quantification of target transcripts regards changes in their expression.

Reference genes are considered “internal controls” or “calibrators” aiming to compare the different samples scrutinized at the same quantitative level, decreasing the probability of occurrence of false positives or negatives in terms of differential gene expression (Amorim *et al*., 2018)⁠. An ideal reference gene must be transcribed stably under several experimental conditions (Dheda *et al*., 2005)⁠. In general, RGs are related to basic cellular activities and metabolic functions, maintaining a constitutive expression under any physiological condition (Yang *et al*., 2014)⁠. However, several studies show that the expression of reference genes may vary according to species, tissues and external factors (Sinha *et al*., 2015; Amorim *et al*., 2018)⁠. Some RGs (such as glyceraldehyde-3-phosphate dehydrogenase, 18S ribosomal RNA, 25S ribosomal, elongation factor 1-alpha) have been widely used. However, they may show statistical variations in transcriptional modulations (Brinkhof *et al*., 2006; Sinha *et al*., 2015)⁠. Therefore, the selection of appropriate RGs is required to normalize RT-qPCR of different samples for reliable results. Given this demand, statistical algorithms such as geNorm (Vandesompele *et al*., 2002)⁠, NormFinder (Andersen *et al*., 2004)⁠ and BestKeeper (Pfaffl *et al*., 2004)⁠ were developed to calculate the stability and reliability of candidate RGs.

The present study provides the first selection and validation of RGs for *C*. *pyramidale* root tissue under salt stress and also associated with arbuscular mycorrhizal fungi (AMF), under controlled conditions. Root samples of control and salt-stressed plants (100 mM NaCl), as well as non-inoculated and inoculated plants (factorial 2x2) at the three times (30 min, 2 h, and 11 days) after exposure to salinity, were analyzed using the three approaches (geNorm, NormFinder, and BestKeeper) to determine the best RGs combination for each experimental condition. Finally, the statistically selected RGs were used to validate the expression of two target transcripts, which were up-regulated in Salt2h RNA-Seq libraries, reinforcing their participation in salinity response.

## Material and Methods

### Plant material and experimental design

The experiments were conducted in the Biosciences Center greenhouse at Federal University of Pernambuco (UFPE) (8°08′58′S, 34°56′55′W) in the Brazilian Northeast region, at an average temperature of 34 ± 2 °C, a relative humidity of 40-60% and 12h/12h of light/dark (maximum light flux: 1400 μmol m^-2^s^-1^). Seeds of *C*. *pyramidale* were provided by Centro de Referência para Recuperação de Áreas Degradadas (CRAD) (Petrolina, Pernambuco, Brazil). The experimental design, the growth conditions, the arbuscular mycorrhizal fungi (AMFs) inoculation process, and physiological data are described in Frosi *et al*. (2018).

⁠In summary, 20-days-old seedlings were transferred to 100 ml pots with sterilized soil. The soil was collected in the Pernambuco state, Brazilian Northeast (7°35′39′S, 34°54′23′W) and was classified as dystrophic yellow Latosol. Seedlings destined for inoculation received soil inoculum with 150 spores from *Acaulospora longula* Spain & N.C. Schenck (URM AMF 07) and *Claroideoglomus etunicatum* (W.N. Becker & Gerd.) C. Walker & A. Schüßler (URM AMF 03), provided by the Mycorrhizas Laboratory’s Inoculum Bank at the Mycology Department of UFPE, totaling 300 spores per plant. Non-inoculated plants received the same amount of autoclaved rhizosphere soil. After 30 days under these conditions, the plants were transferred to pots containing 5 kg of same soil type with a phosphorus (P) concentration adjusted to 33 mg dm^−3^ by applying simple superphosphate (P2O5) in all treatments for standardization. This concentration was determined by Frosi *et al*. (2016), in which 33 mg dm^−3^ of P in the soil promoted higher gains in plant biomass in this species under well-watered conditions.

The plants were kept under hydration (pot capacity - 300 mL) until six months of age, when the salt stress was imposed by saline irrigation with 300 mL of NaCl (100 mM) solution at 08:00 am, every day, for 11 days. This concentration is sufficient to achieve a soil electrical conductivity above 2 mS cm−1, characterizing the soil as saline (US Salinity Laboratory 1954). The 11th day was the maximum stress, considering that the photosynthetic rates were close to zero (see Frosi *et al*., 2018). The experimental design was performed in 2 x 2 factorial (presence or absence of salt and AMF), totaling four treatments: 1) Control (Ct); 2) AMF; 3) Salt and 4) AMF+Salt. When the stress started, the paired collections of salt/inoculated treatments were carried out with their respective controls. The collection times were: 30 min, 2 h, and 11 days after the start of saline irrigation. Roots were harvested from three biological replicates per collection time by treatment (each replicate was represented by one plant), with Ct30min used as Ct2h. Root samples were frozen in liquid nitrogen and stored at -80 °C until RNA isolation. 

### Total RNA Isolation, cDNA synthesis and sequencing

The total RNA was isolated from samples using a modified lithium chloride protocol (Chang *et al*., 1993)⁠. DNA contaminant was removed by treatment with DNase I (Ambion, USA) according to the manufacturer’s instruction. The integrity of total RNA samples was verified by performing 1.5% (w/v) agarose gel electrophoresis, and the quantity and quality of RNA were measured using a Qubit 2.0 Fluorometer (Invitrogen, USA). An aliquot of 0.5 µg of the RNA (of all replicates) was used for the cDNA synthesis using the kit ImProm-IITM Reverse Transcription System (Promega Fitchburg WI, USA), according to the manufacturer’s instructions. The cDNA samples were used as template for RT-qPCR reactions.

Another aliquot of this total RNA (3 µg) was sent to the Center of Functional Genomics (ESALQ/USP, Piracicaba, Brazil) for RNA-Seq libraries synthesis and sequencing. A Bioanalyzer (Agilent) was used to perform a final assessment on the quality prior to RNA-Seq sequencing. The cDNA libraries of control (Ct30min and Ct11d); Salt (30min, 2h, and 11d) and AMF (30min) treatments (three biological replicates from each sample) were generated and sequenced on the Illumina HiSeq 2500 instrument, resulting in 18 RNA-Seq libraries (paired-end, 2 x 100 bp).

### Transcriptome assembly and annotation

After removing adapters, removing low-quality and contaminated sequences, the high-quality [Phred values ≥ 30 (Q30)] reads were subjected to *de novo a*ssembly. The quality of the sequences was evaluated using FastQC0.11.8 tool (Andrews, 2012). For assembling and data analysis, we used the RNA-Seq *de novo* pipeline version 3.1.3 developed by Bourgey *et al*. (2018)⁠. Raw reads were parsed through quality filtration by Trimomatic 0.36 (Bolger *et al*., 2014)⁠ with the following parameters: HEADCROP:13, TRAILING:30, MINLEN:32. Trinity software 2.0.4 was used for our *de novo* transcriptome assembly (Haas *et al*., 2013)⁠. Transdecoder 2.0.1 (https://github.Com/transdecoder/transdecoder/wiki) was used to identify candidate coding regions within the generated transcriptome and to look for open reading frames (ORFs). Trinotate 2.0.2 was used to carry out functional annotation of the transcriptome using the Trinity generated transcriptome file, while transdecoder generated peptide sequence file for final candidate ORFs. Differentially expressed transcripts (for each comparison) were identified by applying the Bioconductor (Huber *et al*., 2015)⁠ program Empirical Analysis of Digital Gene Expression in R (edgeR) (Robinson *et al*., 2009)⁠.

The RNA-Seq data generated in this study were submitted to the Sequence Read Archive (SRA) of NCBI under the accession numbers: PRJNA552047; BioProject: PRJNA552047 and BioSample: SAMN12173571 (https://www.ncbi.nlm.nih.gov/Traces/study/?acc=PRJNA552047). The assembled transcriptome was submitted to the Transcriptome Shotgun Assembly (TSA) of NCBI. This Transcriptome Shotgun Assembly project has been deposited at DDBJ/EMBL/GenBank under the accession GIYP00000000. The version described in this paper is the first version, GIYP01000000.

### Selection of CRGs and primer design

In this study, CRGs were selected based on transcripts with no differential expression in all sequenced libraries (Salt30min, Salt2h, Salt11d, and AMF30min), that is, those with log_2_FC values between 1.0 and -1.0 (p > 0.05; false discovery rate, FDR > 0.05). In order to verify the expression stability of the CRGs, target transcripts (TTs) were selected according to their functional annotation and log_2_FC values > 1.0 associated with p-values and FDRs < 0.05 in at least one of the contrasts (Salt2h).

Primer design was performed using the Primer 3 Plus online tool (http://www.bioinformatics.nl/cgi-bin/primer3plus/primer3plus.cgi), using the following parameters: GC content of 40-60% (ideal content of 50%), annealing temperature of 58-62 °C (ideal of 60 °C), length primer of 18-22 bp and amplicon size of 100-200 bp (Table 1). During primer design, regions with conserved domains/motifs were excluded to prevent primer annealing in multiple regions and, therefore, avoid isoforms amplifications. Additionally, a local BLASTn was performed using Bioedit against the transcriptome analyzed to verify that each primer pair recovered exclusively the desired transcript.

**Table 1 -:** Primer pairs of candidate reference genes (CRGs) and target transcripts (TTs) tested in root tissues of *C*. *pyramidale*.

Gene	Gene description	Sequence (5’ - 3’)	Tm (°C)	GC (%)	Amplicon size (bp)	Slope	E (%)	R2
Reference genes								
*ARP3*	Actin-related protein 3	F:AATTCAGCTCAGCCATCCTTT R:CCAGCCTCACTTTGATTGTTC	60.2 59.7	42.9 47.6	131	3.30	101.01	0.98
*SAC1*	Suppressor of Actin	F:CCAGTCCAGGTCCTTCACATA R:TGAAGAGAATGGTTGGTACGG	60.0 60.0	52.4 47.6	101	3.18	101.14	0.99
*VFB1*	Vier F-box protein1 (At1g47056_)_	F:TCAGCATCACCCACTGTATCA R:GTATTGCCGATGAAGGTTTGA	60.1 60.0	47.6 42.9	169	3.22	104.53	0.97
ATKRS-1	Lysyl-tRNA Synthetase (*At3g11710*)	F:GCATTCTTGCTTATGGTTTCG R:GCTGCTCTCTCATTTCCTTCA	59.7 59.7	42.9 47.6	115	3.33	99.87	0.99
*TUBB2*	Tubulin beta-2 chain	F:GCTGGGTCTAGGCTTGTAGTG R:TCGTGGGAGAAACTACGCTAA	59.0 59.9	57.1 47.6	197	3.53	92.06	0.94
*GRIK2*	Serine/threonine-protein kinase	F:AGAACATTTGGCACGAACAAG R:CTCTCTCAGGAGCCATTGTTG	60.2 60.0	42.9 52.4	120	3.33	100.08	0.98
*MYB4*	Transcription repressor MYB4	F:TCACCGAAAGAGAAGCAGTGT R:AGTCCTATGGCTTGCCTGATT	60.0 60.1	47.6 47.6	185	3.43	95.68	1.0
*CpOXS2*	Zinc finger CCCH domain-containing protein 30	F:CCCAGTTTGACCAAGAATTGA R:CACGCAATGTGGAACCTATTT	60.0 59.9	42.9 42.9	159	3.11	109.67	1.0
*RSH1*	Relat/SPOT homolog1	F:TGACCTCAGCCATGATACCTC R:ATGTTGCGAAGATGATCCAAG	60.1 60.1	42.4 42.9	168	3.51	92.64	0.99
*HAG2*	Histone acetyltransferase type B catalytic subunit	F:GCATGAGACGAGGAGAGTTTG R:CACTTCGCCTGCTTAATTTTG	60.0 59.9	52.4 42.9	108	3.25	102.96	0.99
Target Transcripts								
*RAP2-3*	Ethylene-responsive factor 2	F:TTCTCACTTGCCTCACCTTTG R:GGGTGCTATCATTTCCGACTT	60.4 60.3	47.6 47.6	198	3.49	93.54	0.99
*PIN8*	Putative auxin efflux carrier component 8	F:CTGCCTGAATGATAGCGACTC R:AGAAGCTGATAGCGTGTGGAA	60.0 60.0	52.4 47.6	141	3.32	100.42	0.96

Tm, Melting temperature; E, RT-qPCR amplification efficiencies; R2, Regression coefficient

### RT-qPCR, amplification efficiency, and relative expression

The RT-qPCR reactions were performed on PCR Line Gene 9600 (Bioer Hangzhou Technology, Zhejiang, China) with GoTaq® qPCR Master Mix (Promega, Fitchburg WI, USA). All reactions were prepared in three biological and technical replicates. The reactions were adjusted to 10 μL reaction mixture and consisted of 5 μL of GoTaq® qPCR Master Mix 2x, 1 μL of diluted cDNA (1/10), 0.3 μL of each primer (5 µM) and 3.4 μL of ddH2O and performed as follows: 95 °C for 2 min, followed by 40 cycles of 95 °C for 15 s, 62 °C for 60 s and 72 °C for 15s. After amplification, dissociation curves were produced (60 - 95 °C at a heating rate of 0.1 °C/sec and acquiring fluorescence data at every 0.3 °C) to confirm the specificity of the primers. The amplification efficiency for each primer was calculated from a standard curve generated by cDNA serial dilutions (1/10, 1/100, 1/1000, and 1/10000) in triplicate, according to the following equation: E = 10 (-1/slope of the standard curve) -1. Slopes were considered acceptable in the range of -3.58 to -3.10 for the RT-qPCR assay (Biassoni and Raso, 2014)⁠. The expression pattern of two TTs was performed on Salt30min, 2h, 11d, and AMF30min, using the most suitable RGs as normalizers according to the software used. The Relative Expression Software Tool Rest2009 (REST 2009), a standalone software tool to estimate up and down regulation, was adopted to calculate the relative expression of the TTs. The follow formula was applied: E (ΔCq Target)/ E (ΔCq RG); where E is the average efficiency for each gene; ΔCq regards the difference between mean Cq-value of a control sample and the mean Cq-value of treated sample. This software bases its calculations on pairwise comparisons (target transcripts x reference genes) using randomization and bootstrapping techniques Pairwise Fixed Reallocation Randomization Test© (Pfaffl *et al*., 2002)⁠. Hypothesis testing (p < 0.05) was inferred to determine if there was a significant difference in target transcripts levels between control and treatments. The present work followed the MIQE guidelines (Minimum Information for Publication of Quantitative Real-Time PCR Experiments) (Bustin *et al*., 2009)⁠, aiming to achieve experimental stringency and transparency of data in order to increase the reliability of the results (Table S1).

### Reference genes expression stability

The expression stability of the 10 CRGs (Table 2) was analyzed by three well stablished softwares, geNorm v 3.5 (Vandesompele *et al*., 2002), NormFinder v. 0.953 (Andersen *et al*., 2004), and BestKeeper (Pfaffl *et al*., 2004). For geNorm and NormFinder, the raw Cq-values were transformed into relative quantities-Q = EΔCq, where E represents the average efficiency for each gene, ΔCq is the difference between the lowest quantification cycle (Cq-value) of a sample of a particular gene and the Cq-value of each sample in a dataset (Hellemans *et al*., 2007). Basically, geNorm calculates the expression stability value (M) based on the average of the pairwise variation (V) for each CRG candidate with all genes tested. The M value has a default limit of ≤ 1.5. The lowest M-value indicates the highest stable expression (Vandesompele *et al*., 2002). Besides, the software also provides an estimation of the optimal number of reference genes that must be used for normalization. Based on the geometric mean of the expression of the two most stable CRGs, the normalization factor was calculated by geNorm, and then the NFn+1 with the next most stable gene. Subsequently, the pairwise variation (Vn/n+1) was determined out of two sequential NFs to identify the ideal number of CRGs to be used for a normalization. A normalization factor (NF) is calculated based on the geometric mean of the expression of the two most stable RGs and then the NFn+1 with the next most stable gene. To determine the number of genes to be used for accurate normalization, the pairwise variation (Vn/n+1) was determined out of two sequential NFs (NFn and NFn+1). The cut-off used was V ≤ 0.15, and values below it indicate that an inclusion of an additional RG is not required (Vandesompele *et al*., 2002). 

**Table 2 -:** Expression values (log_*2*_ FC) and statistical analysis (p-value) of 10 reference genes and two target genes of root transcriptome of *C*. *pyramidale* under salt stress and associated with arbuscular mycorrhizal fungi.

Gene	Control x Salt30min		FDR	Control x Salt2h		FDR	Control x Salt11d		FDR	Control x AMF30min		FDR
	log_2_FC	p-value		log_2_FC	p-value		log_2_FC	p-value		log_2_FC	p-value	
References genes												
*ARP3*	0.093	0.91	1.0	-0.058	0.93	1.0	-0.012	0.8	1.0	0.068	0.91	1.0
*SAC1*	-0.015	0.99	1.0	-0.087	0.89	1.0	0.323	0.47	0.84	-0.028	0.96	1.0
*VFB1*	-0.146	0.84	1.0	-0.05	0.93	1.0	0.297	0.49	0.83	-0.026	0.96	1.0
*ATKRS-1*	-0.226	0.76	1.0	0.047	0.93	1.0	0.224	0.57	0.99	0.044	0.93	1.0
*TUBB2*	0.002	1.0	1.0	0.038	0.95	1.0	-0.782	0.06	0.3	0.083	0.88	1.0
*GRIK2*	-0.253	0.75	1.0	-0.347	0.56	1.0	-0.125	0.79	1.0	-0.348	0.56	1.0
*MYB4*	0.012	0.89	1.0	0.04	0.97	1.0	0.308	0.57	0.99	-0.043	0.96	1.0
*CpOXS2*	0.014	1.0	1.0	0.971	0.53	1.0	1.817	0.21	0.56	0.565	0.71	0.99
*RSH1*	0.042	0.99	1.0	0.594	0.48	1.0	-0.694	0.24	0.60	-0.231	0.80	0.93
*HAG2*	0.299	0.71	1.0	0.129	0.84	1.0	-0.012	0.99	1.0	0.364	0.55	0.92
Target transcripts												
*RAP2-3*	0.820	0.26	0.70	11.266	0.00	0.01	-0.147	0.71	0.96	0.311	0.57	0.94
*PIN8*	-0.887	0.66	1.0	6.923	0.00	0.04	-1.438	0.2	1.0	1.12	0.37	0.77

NormFinder uses a mathematical modeling algorithm that considers the intra- and inter-group variation of the CRGs to calculates the stability values. The highest stability is associated with the lowest stability value (Andersen *et al*., 2004).

BestKeeper used the raw Cq-values to calculate the Pearson correlation coefficient (r), obtained by the pairwise comparison between the BestKeeper index generated by the algorithm and the CRGs. This coefficient (r) was used to represent the expression stability, where the CRGs with higher significative (p-value < 0.05) r-value were more stable. Samples with SD-value (standard deviation) > 1 were excluded from analysis. 

The three software, geNorm, NormFinder and BestKeeper, were used to analyze and generate the CRGs stability ranking (Pfaffl *et al*., 2004). For this study, we analyzed three approaches: Global analysis, Salt treatments and AMF assay. In the Global analysis all treatments (Salt, AMF and both) and all collections time (30min, 2h, and 11d) were analyzed together. In the Salt approach, just the plants without inoculation (Salt30min, Salt2h, and Salt11d) were analyzed. For AMF assay, we analyzed all inoculated plants (AMF30min, AMF2h, AMF11d, AMF+Salt30min, AMF+Salt2h, and AMF+Salt11d). These independent approaches were analyzed using each algorithm to verify the most stable RGs for each combination. 

## Results

### Primer specificity, efficiency, and expression profile

Ten transcripts including: Actin-Related Protein (*ARP3*); Suppressor of Actin (*SAC1*); F-Box Proteine (*VFB1*); Lysyl-tRNA Synthetase (*ATKRS-1*); Tubulin (*TUBB2*); Serine/threonine-proteinkinase (*GRIK2*); Transcription repressor (*MYB4*); Zinc finger protein (*CpOXS2*); Relat/SPOT homolog1 (*RSH1*) and Histone acetyltransferase (*HAG2*) were chosen as RGs candidate in RT- qPCR assays of *C*. *pyramidale* roots submitted to salinity and associated with AMF (Table 1). All RGs were considered to have constitutive expression (1.0 < log_2_FC *<* -1.0; p > 0.05 FDR > 0.05) in all sequenced libraries and with known function related to basal or vital cellular processes. In order to validate the selected RGs for RT-qPCR normalization, two up-regulated transcripts, Ethylene-Responsive factor (*RAP2-3*) and Putative Auxin efflux carrier (*PIN8*) in Salt2h (log_2_FC *>* 1; p-value and FDR < 0.05) were used as target transcripts (TTs) (Table 2). 

**Figure 1 -:**
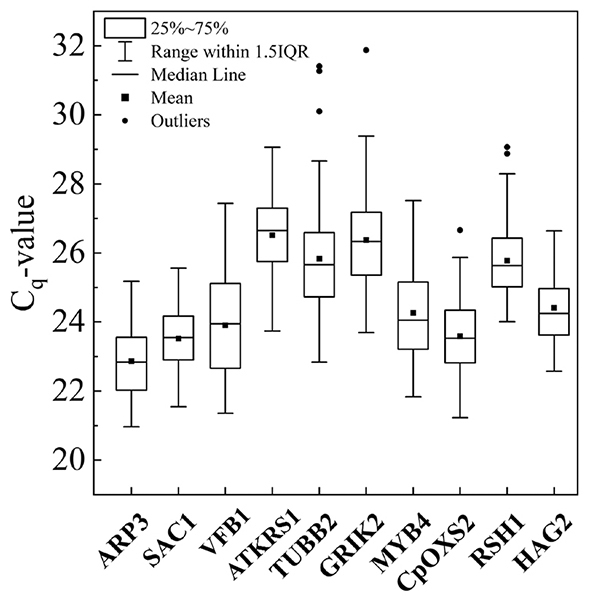
Quantification cycle (Cq-value) of 10 candidate reference genes in root tissues of *Cenostigma pyramidale* under salt stress and associated with arbuscular mycorrhizal fungi (AMF). The Boxplot indicates the interquartile range. The solid horizontal line in the box represents the median value, and the square symbol represents the mean value. The upper and lower lines represent the maximum and minimum values. Dots indicate the outliers.

All primer pairs amplified a single PCR product with the expected size, as indicated by melting curves generated by RT-qPCR (Figure S1). The Tm ranged from 59.0 °C for *TUBB2* to 60.3 °C for *RAP2-3* (Table 1). The amplification efficiencies varied from 92.06 (*TUBB2*) *to* 109.67% (*CpOXS2*), the slope values ranged from 3.11 to 3.53, and the regression coefficients (R2) were higher than 0.94 (Table 1). The Cq average varied from 20.97 (*ARP3*) to 27.88 (*ATKRS-1 a*nd *GRIK2*) (Figure 1; Table S2). This means that *ARP3* and *ATKRS-1* presented the highest and the lowest abundance of transcripts, respectively, for all treatments and time collections.

### Reference genes stability analysis

The expression stability of the potential reference genes was analyzed using three different algorithms: geNorm, NormFinder, and BestKeeper, used to evaluate: (1) Global analysis encompassing all time collections (30min, 2h, and 11d) and treatments (Salt and AMF); (2) Salt treatment(Salt30min, Salt2h, and Salt11d); and (3) AMF assay (AMF30min, AMF2h, AMF11d, AMF+Salt30min, AMF+Salt2h, and AMF+Salt11d). These independent analyses were performed to rank the most stable RGs for each situation, considering each algorithm. 

**Figure 2 -:**
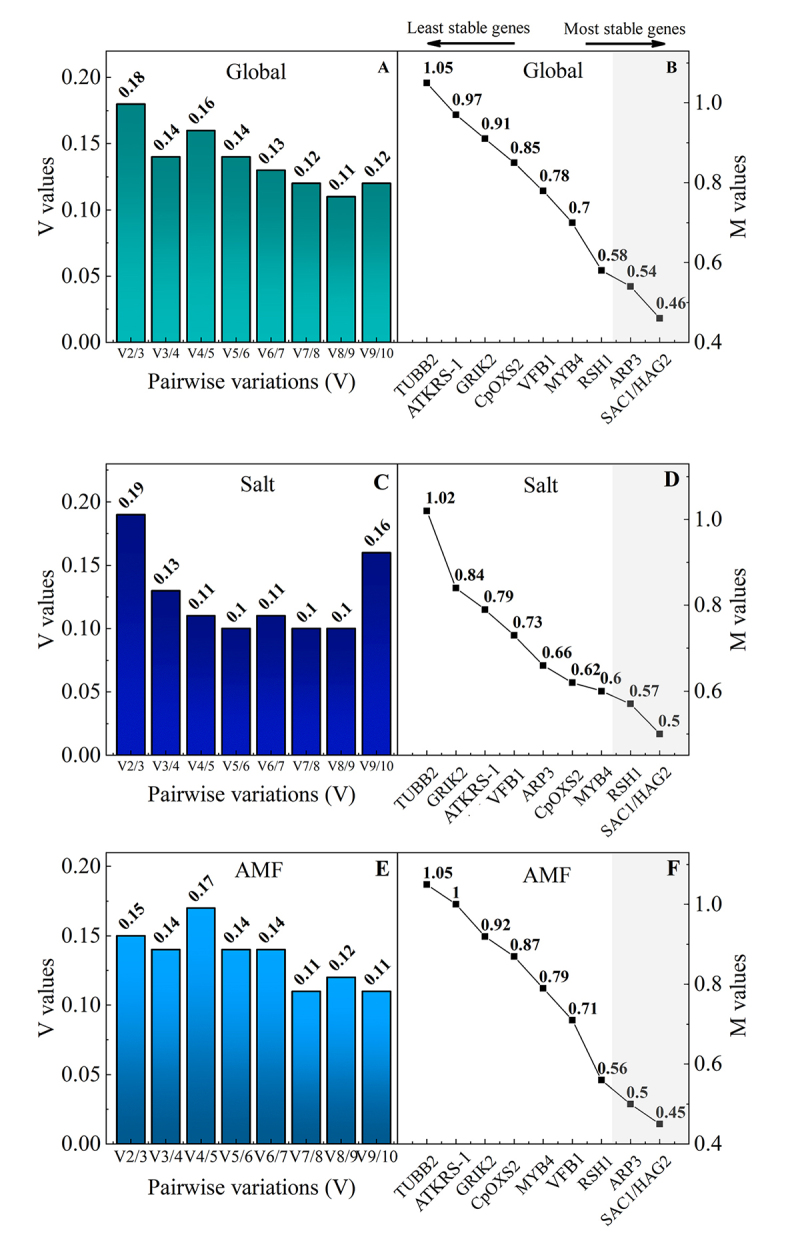
Analysis of reference genes. A, C and E represent the pairwise variation analysis between normalization factors NFn and NFn+1, indicating the optimal number of reference genes (RGs) required for reliable normalization in root tissues of *Cenostigma pyramidale* under salt stress and associated with arbuscular mycorrhizal fungi (AMF) for global, salt and AMF assays, respectively. B, D, and F - average expression stability (M value) of ten candidate reference genes (CRGs) for global, salt, and AMF assays, respectively.

According to the geNorm report, all treatments and CRGs tested showed M values (gene expression stability) (Figures 2B, D, and F) below 1.5. Considering that RGs are not co-regulated, stepwise exclusion of the gene with the highest M value leads to a combination of two RGs with most stable expression values among the tested samples. Thus, the four most stable genes analyzed by geNorm were *HAG2/SAC1*, *aRP3* and *RHS1* for global and AMF conditions, while *HAG2/SAC1*, *RHS1*, and *MYB4* were indicated for Salt assay (Figure 2 B, D, and F). GeNorm was also used to determine the optimal number of RGs necessary for reliable normalization, which was obtained from the “V” value analysis (a V-value below 0.15 indicates that the inclusion of an additional gene is not required for data normalization (Vandesompele *et al*., 2002). For all assays, V-values below 0.15 were obtained in the second analysis (V3/V4), indicating that three RGs are required for normalization of our data via RT-qPCR (Figure 2 A, C, and E).

**Figure 3 -:**
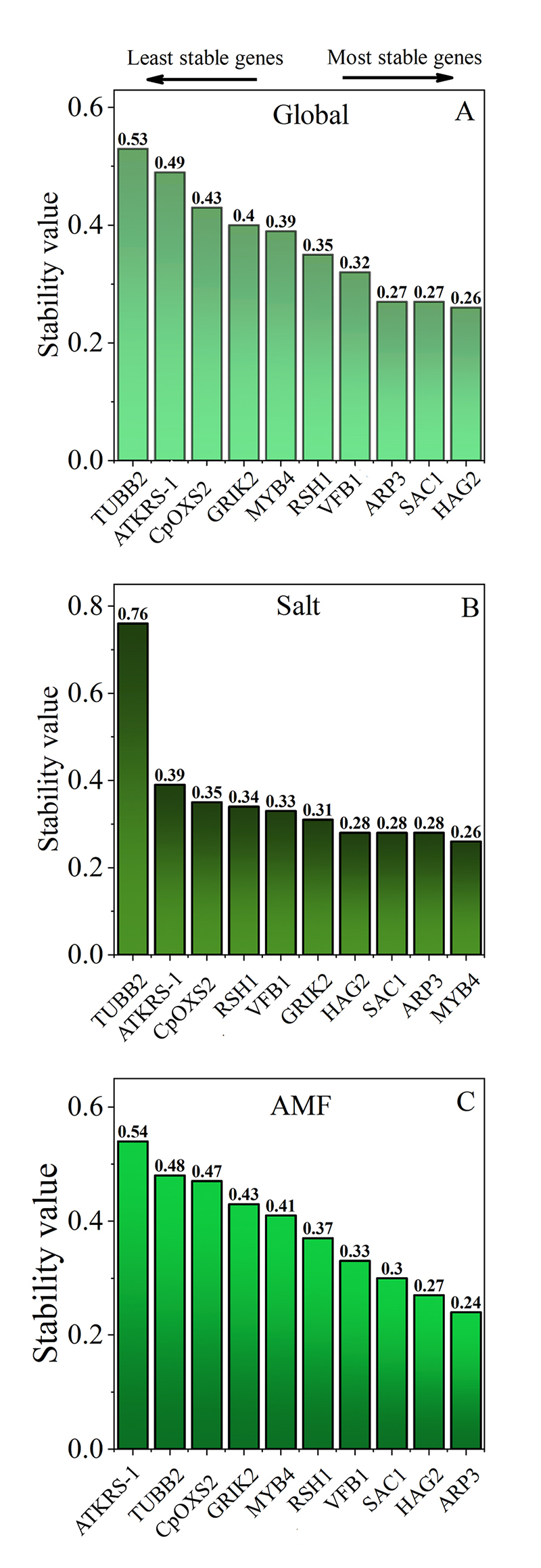
NormFinder stability analysis of 10 candidate reference genes (CRGs) for analyzed assays. Lower values indicate a more stable expression. A- Global (Salt + arbuscular mycorrhizal fungi, AMF); B- Salt; C- AMF assay.

Using NormFinder (Figure 3A), the four most stable RGs for global conditions were *HAG2* (0.26), *SAC1/ARP3* (0.27) and *VFB1* (0.32). For the salt assay (Figure 3B), the most stable RGs were *MYB4* (0.27), *ARP3/SAC1* (0.28) and *HAG2* (0.28). In AMF assay (Figure 3C), the same four RGs were indicated, with changes only in the position: *ARP3* (0.24), *HAG2* (0.27), *SAC1* (0.30) and *VFB1* (0.33).

The BestKeeper algorithm computes the Pearson correlation coefficient to define the BestKeeper index, where the CRGs with SD < 1 and the highest Pearson coefficient of correlation (r) with the BestKeeper index present the highest stability. The global and AMF assays showed the same four most stable genes, with differences only in the second and third positions, where *HAG2* (0.899), *SAC1* (0.884), *ARP3* (*0*.874), and *RSH1* (0.843) were the most stable RGs for the global condition, while *HAG2* (0.87), *ARP3* (0.866), *SAC1* (0.837) and *RSH1* (*0*.818) were selected for the AMF condition (Table 3). For the salt condition, one gene was changed, as compared with the other conditions, where *HAG2* (0.904), *SAC1* (0.899), *MYB4* (0.898) and *RSH1* (0.878) were the four most stable RGs (Table 3).

**Table 3 -:** Ranking of 10 reference genes according BestKeeper software in global time combination of roots of *C*. *pyramidale* under salt stress and associated with arbuscular mycorrhizal fungi (AMF).

Parameters	Reference Genes									
	Global									
	*HAG2*	*SAC1*	*ARP3*	*RSH1*	*CpOXS2*	*ATKRS-1*	*VFB1*	*MYB4*	*GRIK2*	*TUBB2*
*St*andard deviation [± CP]	0.821	0.755	0.775	0.882	0.819	0.925	1.164	1.122	1.077	1.238
Coefficient of variation [% CP]	3.359	3.208	3.389	3.420	3.472	3.487	4.868	4.622	4.085	4.792
Coefficient of correlation [r]	0.899*	0.884*	0.874*	0.843*	0.734*	0.668*	0.902*	0.832*	0.803*	0.731*
	Salt									
	*HAG2*	*SAC1*	*MYB4*	*RSH1*	*ARP3*	*CpOXS2*	*VFB1*	*ATKRS-1*	*TUBB2*	*GRIK2*
*St*andard deviation [± CP]	0.871	0.707	0.883	0.882	0.856	0.893	1.313	1.187	1.499	1.203
Coefficient of variation [% CP]	3.523	2.971	3.563	3.371	3.688	3.786	5.463	4.490	5.691	4.561
Coefficient Of correlation [r]	0.904*	0.899*	0.898*	0.878*	0.875*	0.845*	0.896*	0.895*	0.676*	0.875*
	AMF									
	*HAG2*	*ARP3*	*SAC1*	*RSH1*	*GRIK2*	*CpOXS2*	*ATKRS-1*	*VFB1*	*TUBB2*	*MYB*
*St*andard deviation [± CP]	0.682	0.650	0.664	0.792	0.992	0.741	0.884	1.081	1.051	1.032
Coefficient of variation [% CP]	2.817	2.868	2.843	3.099	3.776	3.147	3.347	4.539	4.116	4.298
Coefficient Of correlation [r]	0.870*	0.866*	0.837*	0.818*	0.772*	0.639*	0.513*	0.916*	0.672*	0.780*

CP = Crossing point; asterisks indicate p<0.05

### Conservation of RGs stability according to algorithm and assay

**Figure 4 -:**
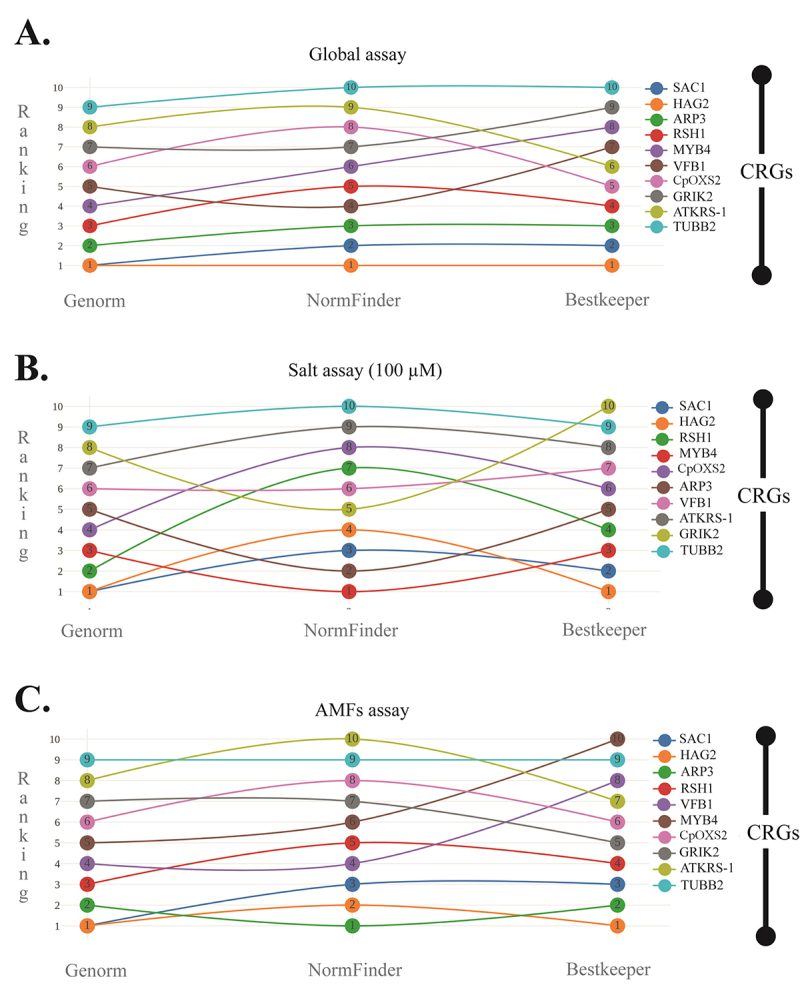
Ranking of 10 candidate reference genes (CRGs) among softwares (geNorm, NormFinder, and BestKeeper). A- Global (Salt + arbuscular mycorrhizal fungi, AMF); B- Salt; C- AMF assay. C.I (95% Confidence Intervals); Std. Error (Standard error); P (H1): Hypothesis Test; UR (up-regulated at p < 0.05); DR (down-regulated at p < 0.05); ns (not significant at the level of p ≤ 0.05)

Considering the three softwares and assays (global, salt, or AMF), there was a small variation among the first four positions (Figure 4). In the global condition, conservation of 100% was observed in the four most stable RGs (*HAG2*, *SAC1*, *aRP3*, and *RSH1*), between geNorm and BestKeeper, with no difference in their positions, while *VFB1 was* the fourth most stably expressed according to NormFinder (showing a 75% conservation with the remaining softwares). By contrast, *TUBB2 was* the least stable RG considering all three softwares (Figure 4A).

Under salinity conditions, geNorm and BestKeeper had 100% conservation of the four most stable RGs (*HAG2*, *SAC*, *RSH1*, and *MYB4*), differing only in their rankings. In NormFinder, only one of the four most stable RGs was different (*ARP3*), maintaining a similarity of 75% with the other softwares (Figure 4B). Except for BestKeeper (which indicated *GRIK2* as the most unstable), *TUBB2* was the least stable gene indicated by geNorm and NormFinder under salinity treatments (Figure 4B).

For AMF assay, there was a conservation of the four most stable RGs based on geNorm and BestKeeper ranking (*HAG2*, *SAC1*, *aRP3*, and *RHS1*), with variations only in their positions. These RGs were the same as for the global condition. In turn, for NormFinder, 75% conservation of these RGs (*ARP3*, *HAG2*, *SAC1*, and *VFB1*) was observed. Regarding the least stable gene, the three softwares diverged, being *TUBB2 ind*icated by geNorm, *ATKRS-1 b*y NormFinder and *MYB4* by BestKeeper (Figure 4C).

Considerable conservation (> 75%) was observed for the four most stable RGs, for each software for the global, salt, and AMF assays. For global and AMF assays, there was a 100% conservation for the four most stable RGs in geNorm (*HAG2*, *SAC1*, *aRP3*, *RSH1*), NormFinder (*HAG2*, *SAC1*, *aRP3*, *VFB1*) and BestKeeper (*HAG2*, *SAC1*, *aRP3*, *RSH1*). Best RGs under salt condition showed 75% conservation with global and AMF assays for geNorm (*HAG2*, *SAC1*, *RSH1*, *MYB4*), NormFinder (*MYB4*, *aRP3*, *SAC1*, *HAG2*) and BestKeeper (*HAG2*, *SAC1*, *MYB4*, *RSH1*). For all conditions, BestKeeper and geNorm indicated the same four most stable RGs (Figure 4).

### Reference genes validation

The expression patterns of two target transcripts, *RAP2-3* and *PIN8* (up-regulated in Salt2h), were evaluated using the three most stable RGs suggested by geNorm: *HAG2*, *SAC1* and *ARP3* for global and AMF assay and *HAG2*, *SAC1 a*nd *RHS1* for salt condition. The up-regulation of *RAP2-3* and *PIN8* transcripts in *C*. *pyramidale* was confirmed by RT-qPCR at Salt2h, with Ct values used to calculate the relative expression by REST software (Tables 2 and 4).

**Table 4 -:** Relative expression calculated by REST software of two target transcripts (RAP2-3 and PIN8) for Salt30min, 2h, 11d and AMF30min in *Cenostigma pyramidale* roots.

_Target transcripts_	Reaction efficiency	Expression	Std. error	C.I	P (H1)	Result
Salt30min						
*CpRAP2-3*	0.93	0.659	0.235-4.027	0.195-4.968	0.787	ns
*Cp PIN8*	1.0	0.23	0.091-0.488	0.066-1.129	0.144	ns
Salt2h						
*CpRAP2-3*	0.93	5.75	3.847-8.842	3.273-11.777	0.0	UR
*Cp PIN8*	1.0	4.49	1.446-11.707	1.317-35.169	0.015	UR
Salt11d						
*CpRAP2-3*	0.93	0.276	0.176 - 0.443	0.124 - 0.608	0.03	DR
*Cp PIN8*	1.0	0.996	0.526 - 2.793	0.278 - 3.389	0.898	ns
AMF30min						
*CpRAP2-3*	0.93	1.506	1.202 - 2.022	0.942 - 2.175	0.101	ns
*Cp PIN8*	1.0	2.457	1.163 - 6.854	0.948 - 7.652	0.105	ns

## Discussion

RT-qPCR is an important approach for detecting and quantifying gene expression, being useful for transcriptomic analysis in different organisms and treatments (Huang *et al*., 2019; Pombo *et al*., 2019)⁠. However, quantification of gene expression is affected by several factors (Maroufi *et al*., 2010)⁠. An accurate data interpretation depends on using appropriate reference genes with minimal expression variations in tissue, treatment, or condition under analysis (Kozera and Rapacz, 2013)⁠. RT-qPCR is currently the most reliable method to validate gene expression results obtained by other high throughput methodologies, such as RNA-Seq. Thus, selecting suitable reference genes is a fundamental requirement to mitigate potential errors in RT-qPCR results (Coêlho *et al*., 2019)⁠. Furthermore, the use of inappropriate RGs may overestimate or underestimate the relative expression of target transcripts (Sinha *et al*., 2015)⁠.

The present work is the first approach related to candidate RGs in *Cenostigma pyramidale*, a native tolerant tree species of the Brazilian semiarid region, in response to salt stress, associated with arbuscular mycorrhizal fungi (AMF), rigorously tested for effective normalization of RT-qPCR data. The expression levels and stability of 10 RGs were evaluated in *C*. *pyramidale* roots under salt stress and inoculated with AMF at different times. To achieve this goal, we used geNorm, NormFinder and BestKeeper tools to analyze the stability of RGs in all treatments and times separately and together (global condition). Mycorrhizal colonization of plants has been studied because it promotes increases in plant growth, water, and mineral nutrient uptake, and increases plant stress tolerance under abiotic stress (Frosi *et al*., 2016, 2018)⁠. However, few studies have been conducted regarding the stability of RGs during mycorrhizal colonization, necessary for accurate and reliable normalization of RT-qPCR (Fuentes *et al*., 2016)⁠.

In our study, a 100% conservation of CRGs among the four most stable genes was pointed out by geNorm, NormFinder, and BestKeeper, for global and AMF conditions (Figures 3 and 4; Table 3). The optimal number of RGs necessary for reliable normalization was three according to geNorm, namely *HAG2*, *SAC1*, *aRP3* (Figure 2 A, B, E and F). We obtained a convergence of 75% of these four RGs for each software under salt condition compared with global and AMF conditions. Regarding the number of adequate genes to be used for normalization by geNorm for salt conditions, *HAG2*, *SAC1*, *RSH1* regarded the best combination (Figure 2 C and D). The RGs stability rankings often were not entirely identical for the same experimental condition, due to distinct statistical algorithms and analytical procedures for each software (Yang *et al*., 2015)⁠.

Reference genes obtained in our study are involved in basic cell functions. Histone acetyltransferase type B catalytic subunit (*HAG2*) promotes histone modification process, which plays a critical role in regulating gene expression (Pfluger and Wagner, 2008)⁠. Acetylation by histone acetyltransferases is generally correlated with increased gene activity (Chen, 2007)⁠. An extensive series of biochemical fractionation studies identified type B histone acetyltransferases as a distinct class of enzymes that can be found in the cytoplasm and, most importantly, with the ability to acetylate free (not nucleosomal) histones (Parthun, 2007)⁠. *HAG2* is a multisubunit complex responsible for acetylating newly synthesized histone H4 on the lysines K5 and K12 (Haigney *et al*., 2015)⁠. Histone acetylation plays essential roles in regulating plant cell cycle, flowering time, response to environmental conditions, hormone signals, and epigenetic processes (Chen, 2007)⁠.

The second reference gene encodes a phosphoinositide phosphatase (*SAC1*; Suppressor of Actin 1), which regulates the levels of phosphatidyl inositol phosphates due to its phosphatase activity. In general, it is located in the endoplasmic reticulum (ER) and Golgi apparatus (GA) (Strahl and Thorner, 2007)⁠. A study performed by Despres *et al*. (2003)⁠ verified that three AtSAC1 proteins are located exclusively in the ER. Other phosphatases may regulate plant phosphoinositide pools in the GA. Their function was deduced based on similarity to the *Saccharomyces cerevisiae* SAC1 protein, which regulates the phosphatidylinositol 4-phosphate pool. Phosphoinositides are ubiquitous membrane lipids, and they play important roles in cellular processes like membrane trafficking and organization of the cytoskeleton, such as actin (Despres *et al*., 2003; De Craene *et al*., 2017)⁠. In higher plants, these lipids are also believed to be of great importance for signal transduction (Despres *et al*., 2003)⁠. There is evidence that plant inositol phospholipids play a role in the Ca^2+^ signaling pathway (DeWald, 2001). Due to its function, Hsu and Mao (2013)⁠ reported that Sac1 plays an essential housekeeping role in multicellular organisms.

The other reference gene established in our study for global and AMF condition was an actin-related protein 3 (*ARP3*). The organization and function of the actin cytoskeleton are regulated by several actin-binding proteins, including profilin, actin-depolymerizing factor, formin, and the Actin-Related Protein 2/3 (ARP2/3) complex (Staiger and Blanchoin, 2006)⁠. Arabidopsis ARP2/3 complex subunits have also been reported to be involved with the stomatal movement  (Li *et al*., 2014)⁠, salt stress (Zhao *et al*., 2013)⁠ and root hair development (Mathur, 2003)⁠. Root hair growth under certain conditions is disturbed in Arp2/3 mutants. *ARP3* is also part of the ARP3/DISTORTED1 (DIS1) complex that plays different roles in gravitropism and phototropism (Reboulet *et al*., 2010)⁠. ARP3/DIS1 takes part in amyloplastic sedimentation by affecting local apparent viscosity in the central columella cells and in asymmetric auxin redistribution across the root tips through the modulation of auxin efflux carriers, such as PIN cycling (Zou *et al*.,2016)⁠. Biochemical, genetic, and functional studies have revealed that actin-related proteins play divearse and significant roles in processes of vesicle motility, mitosis, actin filament dynamics, and modulation of chromatin structure. They are related to basic cell functions (Muller *et al*., 2005)⁠.

Putative GTP diphosphokinase, chloroplastic (*RSH1*) *wa*s indicated by geNorm for salt condition normalization. Several studies have shown that plant RSH proteins are predominantly located in chloroplasts and plastids (Boniecka *et al*., 2017)⁠. This gene is involved with a fast plant (p)ppGpp (guanosine tetraphosphate/pentaphosphate) regulation-mediated response to pathogen attack and other stresses. It belongs to the RelA/SpoT family (Boniecka *et al*., 2017)⁠. RSH1 seems to function as the major (p)ppGpp hydrolase in plants (Sugliani *et al*., 2016)⁠. The *AtRSH* genes are the first eukaryotic homologs of bacterial RelA/SpoT genes described to date (van der Biezen *et al*., 2000)⁠. Although (p)ppGpp appears to contribute to normal plant growth and development (Sugliani *et al*., 2016)⁠, it may also have stress-related functions. Levels of (p)ppGpp have been shown to increase in response to abiotic stress and to stress-related plant hormones (Ihara *et al*., 2015). However, in our study, this gene exhibited a constitutive expression pattern, being a suitable RG for *C*. *pyramidale* under the analyzed conditions.

For validation in all sequenced libraries (Salt30min, 2h, 11d, and AMF30), the *HAG2*, *SAC1* and *ARP3* were used to normalize the expression of two target transcripts up-regulated in Salt2h: ethylene-responsive transcription factor (*RAP2-3*) and Putative auxin efflux carrier component 8 (*PIN8*).

*RAP2-3* belongs to the superfamily AP2/ERF and the subfamily ERF. Transcription factors interact with cis-elements in the promoter regions of stressed-genes, inducing the expression of several genes and resulting in tolerance to stress (Wu *et al*., 2014)⁠. Specifically, AP2/ERF is one of the largest plant transcription factor families participating in plant development and resistance to biotic and abiotic stresses. It has been characterized based on either one or two AP2 domains, where the ERF subfamily has one AP2 domain (Pan *et al*., 2019)⁠. Plant hormones as salicylic acid (SA), methyl-jasmonate acid (MeJA), 1-aminocyclopropanecarboxylic acid (ACC), and abscisic acid (ABA), besides abiotic (including NaCl) and biotic stresses, could induce the expression of *RAP2-3* gene (Pan *et al*., 2019)⁠. In addition, Papdi *et al*. (2015) observed in *A*. *thaliana* that the stabilized RAP2 transcription factors, including *RAP2-3*, *ca*n prolong the ABA-mediated activation of a subset of osmotic responsive genes.

The other target transcript tested in our study was a putative auxin efflux carrier component 8 (*PIN8*). The spatio-temporal control of auxin levels is fundamental for developmental events such as organ initiation, embryogenesis, and root differentiation. PIN8 (as PIN5 and PIN6) is predominantly located in the endoplasmic reticulum, although plasma membrane location cases have also been reported for PIN5 and PIN8 (Ganguly *et al*., 2014)⁠. PIN5, 6 and 8 are not directly involved with the cell-to-cell auxin transport but play a role in the intracellular regulation of auxin homeostasis by working together with members of the PIN-LIKE auxin efflux carriers (Barbez *et al*., 2012)⁠. In *Arabidopsis*, PIN proteins are involved with the crosstalk of auxin, ethylene, cytokinin, and strigolactone in root development and abiotic stresses (Wang *et al*., 2009; Shen *et al*., 2010; Yue *et al*., 2015)⁠. The capacity of PIN8 to change the composition of the auxin pool and to limit auxin availability for transcriptional gene regulation was evidenced in tobacco plants and Arabidopsis using dominant interference by overexpression (Dal Bosco *et al*., 2012)⁠. The PIN genes have been reported to play a crucial role in plants adapted the growth environment by response to several abiotic stress, such as salt stress (Habets and Offringa, 2014). Hu *et al*. (2021) observed the up-regulation in *PIN8* along drought stress, including the time collection of 48 h, in leaves of 3 months-old *Liriodendron chinense*. Our study brings the new insight of *PIN8* related to salt stress in the root tissue.

## Conclusion

We have tested the expression stabilities of 10 candidate reference genes (CRGs) required for reliable normalization in root tissues of an important woody legume tree representative of semiarid areas - *Cenostigma pyramidale*. The CRGs were tested in different experimental conditions, including salt stress and associated with arbuscular mycorrhizal fungi. Based on this data, for global and AMF assays *HAG2*, *SAC1*, and *ARP3* are recommended, while salt stress assay *HAG2*, *SAC1*, and *RHS1* are the most suitable reference genes for normalization of expression analysis. Our results highlight the importance of selecting and validating reliable reference genes for an accurate interpretation of transcriptomic data. Also, depending on the treatments analyzed, the most reliable reference genes may change.

## Supplementary material

The following online material is available for this article:

Table S1 - MIQE checklist for authors, reviewers and editors.

Table S2 - Cq values obtained from reference genes.

Figure S1- Melting temperature of ten candidates reference genes (RGs) and two target transcripts (TTs).
