# Supplementary material for: Reference genes for quantitative real-time PCR normalization of Cenostigma pyramidale roots under salt stress and mycorrhizal association
Source: Genet Mol Biol. 2021 May 31;44(2):e20200424. doi: 10.1590/1678-4685-GMB-2020-0424 (PMC8167929; doi:10.1590/1678-4685-GMB-2020-0424)
Supplement: Supplementary file 1 [file 1415-4757-GMB-44-2-e20200424-s1.pdf]

Genetics and Molecular Biology
https://doi.org/10.1590/1678-4685-GMB-2020-0424

Supplementary Material to "Reference genes for quantitative real-time PCR normalization of *Cenostigma pyramidale* roots under salt stress and mycorrhizal association"

Table S1 - MIQE checklist for authors, reviewers and editors. All essential information (E) must be submitted with the manuscript. Desirable information (D) should be submitted, if available.

ITEM TO CHECK	Importance	Checklist	Yes/No/Not Applicable (NA)	DESCRIPTION
EXPERIMENTAL DESIGN				
Definition of experimental and control groups	E	ok	Yes	The control was maintained under constant hydration, with no association with arbuscular mycorrhizal fungi (AMF); The treatments consisted of saline solution irrigation (NaCl, 100mM) and inoculation with AMF The collection of roots was performed during the stress times employed (30min, 2h and 11 days) after the irrigation with saline solution. For evaluating candidate reference genes (CRGs), qPCR reactions of all treatments and controls were analyzed. After RGs selection for Global (all treatments), Salt (only saline treatments) and AMF (only inoculated treatments) assays, two target genes were scrutinized and validated by qPCR in Salt30min, Salt2h, Salt11d and AMF30min treatments.
Number within each group	E	ok	Yes	Three biological replicates (RB) were used for each treatment (one RB = one plant).
Assay carried out by core lab or investigator's lab?	D	ok	Yes	Investigator's lab.
Acknowledgment of authors' contributions	D	ok	Yes	Conceived and designed the experiments: GF, MGS, LCM, AMBI, VP;<br>Performed the experiments and analyzed the data: GF, JRCFN, VP, LLL, SS, JPBN;<br>Contributed reagents / materials / analysis tools: AMBI, EAK. LCM, MGS;<br>Wrote the manuscript: GF, JCRFN, AMBI, MGS, VP. MGS, AMBI and LCM coordinated the research project.
SAMPLE				
Description	E	ok	Yes	Each biological sample consisted of *Cenostigma pyramidale* roots.<br>Seeds were sterilized in 1% hypochlorite (v/v)/5 min, washed with deionized water and placed in trays containing sterilized washed sand to germinate. After 20 days, seedlings were transferred to 100 ml pots with sterilized soil. Seedlings destined for inoculation received soil inoculum (150 spores from each AMF in roots = 300 spores/ plant). Non-inoculated plants received the same amount of autoclaved rhizosphere soil. After 30 days under these conditions, the plants were transferred to pots containing 5 kg of same soil type with a phosphorus (P) concentration adjusted to 33 mg dm -3 by applying simple superphosphate (P2O5) in all treatments for standardization. Salt stress was imposed by irrigation with a NaCl 100 mM solution (300 ml) around 08:00 am every day when plants completed 6 months of development. (see Material and Methods section and Frosi et al. 2018, as cited in this topic).
Volume/mass of sample processed	D	–	NA	–
Microdissection or macrodissection	E	–	NA	–

ITEM TO CHECK	Importance	Checklist	Yes/No/Not Applicable (NA)	DESCRIPTION
Processing procedure	E	Ok	Yes	Upon collecting root tissues, each sample was packed in aluminum foil and then dipped in liquid nitrogen. Next, the samples were immediately stored in a deep freezer -80°C
If frozen - how and how quickly?	E	ok	Yes	The root tissue was collected from the control, and from each treatment performed and immediately dipped in liquid nitrogen.
If fixed - with what, how quickly?	E	–	NA	–
Sample storage conditions and duration (especially for FFPE samples)	E	ok	Yes	Samples stored at -80ºC for five months before being processed for RNA extraction
NUCLEIC ACID EXTRACTION				
Procedure and/or instrumentation	E	ok	Yes	Lithium chloride method (Chang et al., 1993)
Name of kit and details of any modifications	E	-	NA	-
Source of additional reagents used	D	–	NA	–
Details of DNase or RNAse treatment	E	ok	Yes	SV Total RNA Isolation System protocol (Promega)
Contamination assessment (DNA or RNA)	E	ok	Yes	Using the ratio of 260 nm/280 nm.
Nucleic acid quantification	E	ok	Yes	Qubit (Invitrogen, USA).
Instrument and method	E	ok	Yes	Fluorometry and spectrophotometric analysis (at 280 nm and 260 nm) as well as by agarose gel electrophoresis
Purity (A260/A280)	D	ok	Yes	1.8 < Purity < 2.0
Yield	D	-	Yes	Variable, depending on the treatment investigated. Information available upon request.
RNA integrity method/instrument	E	ok	Yes	Gel electrophoresis at 1.5% agarose and fluorimetry (Qubit, Oregon, USA).
RIN/RQI or Cq of 3' and 5' transcripts	E	–	No	–
Electrophoresis traces	D	ok	Yes	Pictures are available upon request.
Inhibition testing (Cq dilutions, spike or other)	E	ok	Yes	Using the formula $E\% = [-1 + 10^{(-1/slope)}] \times 100$ and testing dilution series of sample cDNA, incorporating several orders of magnitude (100 – 0.1 ng) and C_q values were plotted against log_{10}[cDNA quantity].
REVERSE TRANSCRIPTION				
Complete reaction conditions	E	ok	Yes	For each sample, 0.5 µg of the RNA was reversed-transcribed into cDNA using the Improm-IITM Reverse Transcriptional System (Promega) with oligo(dT) primers following the manufacture's recommendations.
Amount of RNA and reaction volume	E	ok	Yes	0.5 µg of total RNA per 20 µL reaction volume for each reverse transcription reaction.
Priming oligonucleotide (if using GSP) and concentration	E	–	NA	–
Reverse transcriptase and concentration	E	ok	Yes	The ImProm-II™ Reverse Transcriptase System (Ref. A3800) supplied with this system is formulated for efficient first-strand cDNA synthesis or RT-PCR applications (it was used 1µL enzyme/ 20 µL reaction).
Temperature and time	E	ok	Yes	42°C for 60 min.
Manufacturer of reagents and catalogue numbers	D	ok	Yes	See ImProm-II™ Reverse Transcription System protocol (link: https://www.promega.com/~/media/files/resources/protocols/technical%20manuals/0/improm-ii%20reverse%20transcription%20system%20protocol.pdf)
Cqs with and without RT	D	ok	Yes	
Storage conditions of cDNA	D	ok	Yes	Permanently at -20 °C in sterile test tubes.
qPCR TARGET INFORMATION				
If multiplex, efficiency and LOD of each assay.	E	–	NA	–

ITEM TO CHECK	Importance	Checklist	Yes/No/Not Applicable (NA)	DESCRIPTION
Sequence accession number	E	ok	Yes	See Table 2 for the sequence accession numbers of the RGs and target genes.
Location of amplicon	D	ok	Yes	Preferably distinct exons Table 1
Amplicon length	E	ok	Yes	101 to 198 nt.
In silico specificity screen (BLAST, etc.)	E	ok	Yes	Used local BLAST against *de novo* root transcriptome of *Cenostigma pyramidale* as target organism (data not show in the manuscript).
Pseudogenes, retropseudogenes or other homologs?	D	ok	Yes	Pseudogenes were excluded from the analysis.
Sequence alignment	D	-	No	Alignment available upon request.
Secondary structure analysis of amplicon	D	–	No	-
Location of each primer by exon or intron (if applicable)	E	ok	Yes	Care was taken to have the primer sequence spanning exon boundaries (Table 1).
What splice variants are targeted?	E	ok	Yes	No splice variants were targeted because there is no information about them in the analyzed organism.
qPCR OLIGONUCLEOTIDES				
Primer sequences	E	ok	Yes	See Table 1
RTPrimerDB Identification Number	D	–	NA	-
Probe sequences	D	–	NA	-
Location and identity of any modifications	E	–	NA	No modifications.
Manufacturer of oligonucleotides	D	ok	Yes	Macrogen – Korea (https://dna.macrogen.com/eng/)
Purification method	D	ok	Yes	Desalted.
qPCR PROTOCOL				
Complete reaction conditions	E	ok	Yes	The reactions were performed on PCR Line Gene 9600 (Bioer Hangzhou Technology, Zehjiang, China) using GoTaq® qPCR Master Mix (Promega, Fitchburg WI, USA). All reactions were performed in three biological and technical replicates. The reactions were prepared in a 10 uL reaction mixture consisted of 5 uL of GoTaq® qPCR Master Mix 2x, 1 uL of diluted cDNA (1/10), 0.3 uL of each primer (5uM) and 3.4 uL of ddH2O.
Reaction volume and amount of cDNA/DNA	E	ok	Yes	20 ng of cDNA in 10 µL reaction volume.
Primer, (probe), Mg++ and dNTP concentrations	E	ok	Yes	Final concentration of 500 nM for each primer in the reaction, 2.5 mM $MgCl_2$ and dNTP concentration provided with the Master mix (proprietary information).
Polymerase identity and concentration	E	ok	Yes	GoTaq®Hot Start Polymerase [GoTaq® qPCR Master Mix, 2x].
Buffer/kit identity and manufacturer	E	Ok	Yes	GoTaq® qPCR Master Mix, 2x (Promega, Fitchburg WI, USA).
Exact chemical constitution of the buffer	D	ok	Yes	dsDNA-binding dye, BRYT Green® Dye, a low level of carboxy-X-rhodamine (CXR) reference dye (identical to ROX™ dye), GoTaq®Hot Start Polymerase, MgCl2, dNTPs. GoTaq® qPCR Master Mix, 2x (Promega, Fitchburg WI, USA).
Additives (SYBR Green I, DMSO, etc.)	E	-	No	
Manufacturer of plates/tubes and catalog number	D	ok	Yes	Sapphire microplate 96-Well Reaction Plate, Cat No.: 652290.
Complete thermocycling parameters	E	ok	Yes	95°C for 2 min as an initial step followed by 40 cycles of 95 °C for 15 s, 58 °C for 60 s and 72º for 15 s . After amplification, dissociation curves were produced (60 °C to 95 °C at a heating rate of 0.1 °C/sec and acquiring fluorescence data every 0.3 °C) to discriminate the main reaction products from other nonspecific ones or primer-dimers.
Reaction setup (manual/robotic)	D	ok	Yes	Manual
Manufacturer of qPCR instrument	E	ok	Yes	LineGene 9660 model (Bioer).

ITEM TO CHECK	Importance	Checklist	Yes/No/Not Applicable (NA)	DESCRIPTION
**qPCR VALIDATION**				
Evidence of optimization (from gradients)	D	–	No	No optimization steps were realized.
Specificity (gel, sequence, melt, or digest)	E	ok	Yes	Melting curves for each biological replicate (see Figure 1).
For SYBR Green I, Cq of the NTC	E	ok	Yes	See Table S2
Standard curves with slope and efficiency	E	ok	Yes	See Table 1.
PCR efficiency calculated from slope	E	ok	Yes	See Table 1.
Confidence interval for PCR efficiency or standard error	D	–	–	-
r2 of standard curve	E	ok	Yes	> 0.94
Linear dynamic range	E	ok	Yes	Average 22.86 to 26.52 Cycles.
Cq variation at lower limit	E	ok	Yes	Standard deviation=0.92.
Confidence intervals throughout range	D	–	–	-
Evidence for limit of detection	E	–	–	-
If multiplex, efficiency and LOD /each assay.	E	–	NA	-
**DATA ANALYSIS**				
qPCR analysis program (source, version)	E	ok	Yes	LineGene 9660 Software (Bioer).
Cq method determination	E	ok	Yes	Manual, set for all assays at ΔRn=0.25.
Outlier identification and disposition	E	ok	Yes	None of Cq values was discarded
Results of NTCs	E	ok	Yes	No amplification.
Justification of number and choice of reference genes	E	ok	Yes	Although statistical data from the geNorm software indicate three reference genes as suitable to obtain a normalization factor, three reference genes were used. Such quantitative was employment following the instructions of Vandesompele et al. (2002).
Description of normalization method	E	ok	Yes	Three strategies (geNorm, NormFinder and BesKeeper) were used to indicate the reference genes used in the present study.
Number and concordance of biological replicates	D	ok	Yes	There were 3 biological replicates of each analyzed treatment.
Number and stage (RT or qPCR) of technical replicates	E	ok	Yes	There were 3 technical replicates for each biological replicate.
Repeatability (intra-assay variation)	E	ok	Yes	Dependent on the analyzed transcript.
Reproducibility (inter-assay variation, %CV)	D	ok	Yes	95% Confidence Intervals, Slope= -3.11 to -3.53.
Power analysis	D	–	No	-
Statistical methods for result significance	E	ok	Yes	For the reference gene indication, the statistical methods were contained in the geNorm, NormFinder and BestKeeper algorithms. For relative expression analysis: REST2009 software ($p<0.05$).
Software (source, version)	E	ok	Yes	See Material and Methods.
Cq or raw data submission using RDML	D	ok	Yes	See Table S2 .

Legend for abbreviations: NA= not applicable.
