# Supplementary material for: Reference genes for quantitative real-time PCR normalization of Cenostigma pyramidale roots under salt stress and mycorrhizal association
Source: Genet Mol Biol. 2021 May 31;44(2):e20200424. doi: 10.1590/1678-4685-GMB-2020-0424 (PMC8167929; doi:10.1590/1678-4685-GMB-2020-0424)
Supplement: Supplementary file 2 [file 1415-4757-GMB-44-2-e20200424-s2.pdf]

Genetics and Molecular Biology
https://doi.org/10.1590/1678-4685-GMB-2020-0424

Supplementary Material to "Reference genes for quantitative real-time PCR normalization of *Cenostigma pyramidale* roots under salt stress and mycorrhizal association"

Table S2 - Cqs obtained for the candidate reference genes analyzed for the salt stress assay in roots of *Cenostigma pyramidale*.

**Treatment**	BR/TR	RG 1	RG 2	RG 3	RG 4	RG 5	RG 6	RG 7	RG 8	RG 9	RG 10
		ARP3	***SAC1***	***VFB1***	***ATKRS-1***	***TUBB2***	***GRIK2***	***MYB4***	***CpOXS2***	***RSH1***	***HAG2***
Ct30m	1	22.40	23.89	25.32	27.27	26.46	25.99	24.68	23.65	25.94	24.68
Ct30m	1	23.35	23.73	25.45	27.13	25.66	25.91	25.36	23.69	26.33	24.51
Ct30m	1	23.39	23.38	24.77	27.08	26.07	25.87	25.13	23.25	25.91	24.83
Ct30m	2	23.32	23.66	25.46	27.23	28.53	27.22	24.77	23.55	26.42	23.90
Ct30m	2	23.65	23.59	25.27	27.66	27.79	27.59	25.11	23.02	26.43	24.66
Ct30m	2	23.03	23.90	25.12	27.61	26.98	26.70	25.02	23.37	26.11	24.34
Ct30m	3	23.19	22.66	23.32	25.43	27.97	25.20	24.09	22.54	25.14	23.51
Ct30m	3	22.88	23.28	22.55	25.18	28.67	25.42	23.99	22.68	25.34	24.77
Ct30m	3	23.17	23.66	22.73	25.43	27.94	25.50	24.79	22.35	25.14	23.99
Salt30m	1	22.19	22.81	22.43	24.28	25.03	24.12	23.44	21.53	25.49	24.16
Salt30m	1	22.11	22.23	22.05	24.56	23.81	24.28	22.73	21.23	25.34	23.51
Salt30m	1	21.90	22.68	21.36	23.91	24.51	23.69	23.11	22.16	25.36	23.39
Salt30m	2	23.60	24.54	25.39	26.83	26.04	25.81	25.39	24.17	26.91	25.17
Salt30m	2	24.38	24.91	25.00	26.24	26.14	26.73	25.62	24.53	26.44	25.72
Salt30m	2	24.54	24.42	24.49	26.88	25.59	26.33	25.40	24.83	27.49	25.93

**Treatment**	BR/TR	RG 1<br>***ARP3***	RG 2<br>***SAC1***	RG 3<br>***VFB1***	RG 4<br>***ATKRS-1***	RG 5<br>***TUBB2***	RG 6<br>***GRIK2***	RG 7<br>***MYB4***	RG 8<br>***CpOXS2***	RG 9<br>***RSH1***	RG 10<br>***HAG2***
Salt30m	3	24.02	25.56	26.38	28.89	25.86	27.50	26.82	24.82	28.06	26.57
Salt30m	3	24.04	25.49	25.88	27.77	26.28	27.18	27.52	24.42	28.29	26.12
Salt30m	3	23.69	24.59	25.52	29.06	26.41	31.88	27.29	25.25	27.68	26.44
AMF30m	1	22.99	24.89	22.66	25.05	26.49	26.49	23.88	22.69	26.43	24.54
AMF30m	1	22.91	23.69	22.63	25.54	26.33	25.17	23.47	22.80	26.05	24.07
AMF30m	1	22.26	23.71	22.88	25.63	25.30	25.35	24.05	22.20	25.67	24.79
AMF30m	2	23.98	24.23	25.28	26.54	27.22	26.70	24.78	23.52	25.91	25.03
AMF30m	2	22.94	23.66	25.30	26.53	27.25	26.90	24.72	23.38	25.83	24.66
AMF30m	2	22.67	23.55	25.49	26.82	27.32	26.54	24.76	24.03	25.70	24.30
AMF30m	3	23.06	23.88	23.77	26.14	25.31	25.93	24.46	23.69	25.73	24.30
AMF30m	3	23.05	23.21	23.15	26.08	24.37	26.32	24.39	23.03	26.17	24.06
AMF30m	3	23.06	23.55	23.46	26.11	24.84	26.13	24.43	23.36	25.95	24.18
AMF+Salt30m	1	23.21	23.60	24.46	27.70	25.62	25.46	23.48	23.31	26.73	24.18
AMF+Salt30m	1	23.66	24.32	24.44	26.65	25.95	25.90	23.21	23.54	25.94	24.53
AMF+Salt30m	1	22.96	23.96	23.62	26.52	25.43	25.59	23.70	24.07	26.00	24.72
AMF+Salt30m	2	22.70	23.49	23.31	26.08	24.77	24.88	23.21	22.18	25.54	23.86
AMF+Salt30m	2	22.89	23.06	22.52	25.44	24.95	25.26	23.45	22.89	25.01	23.62
AMF+Salt30m	2	21.99	22.79	22.45	24.83	24.29	24.84	22.80	22.48	25.55	23.87
AMF+Salt30m	3	22.05	22.26	22.32	25.17	24.39	24.49	22.99	21.91	24.53	23.28
AMF+Salt30m	3	21.97	22.47	22.39	25.26	24.96	24.67	22.86	21.90	24.24	23.72
AMF+Salt30m	3	21.46	22.57	22.23	25.17	24.76	24.62	22.63	21.95	24.64	23.44
Salt2h	1	23.83	25.24	26.03	28.04	31.27	29.03	25.05	24.56	27.29	25.81
Salt2h	1	24.40	25.20	25.29	28.61	30.10	28.55	25.64	24.40	27.22	25.84
Salt2h	1	23.89	25.01	25.33	27.72	31.41	28.14	25.78	24.65	26.58	26.30
Salt2h	2	23.56	24.62	25.40	27.14	27.77	26.39	25.37	24.08	26.17	26.06

**Treatment**	BR/TR	RG 1 ***ARP3***	RG 2 ***SAC1***	RG 3 ***VFB1***	RG 4 ***ATKRS-1***	RG 5 ***TUBB2***	RG 6 ***GRIK2***	RG 7 ***MYB4***	RG 8 ***CpOXS2***	RG 9 ***RSH1***	RG 10 ***HAG2***
Salt2h	2	23.74	23.88	25.41	26.36	26.42	27.04	25.04	23.75	25.36	25.33
Salt2h	2	23.94	24.15	25.45	26.97	27.54	26.70	25.02	24.34	26.81	25.59
Salt2h	3	22.28	22.97	22.32	25.95	26.72	26.10	24.05	22.73	25.66	24.00
Salt2h	3	22.63	22.90	22.51	26.02	26.10	24.62	23.26	22.91	25.56	24.25
Salt2h	3	22.46	22.94	22.42	25.99	26.41	25.36	23.66	22.82	25.61	24.13
AMF2h	1	22.45	22.87	22.75	28.19	25.73	25.47	23.78	23.09	25.81	23.93
AMF2h	1	22.50	22.91	23.46	26.69	26.17	26.37	22.79	23.20	25.63	23.74
AMF2h	1	22.69	23.23	23.72	26.52	26.28	25.49	23.65	23.32	24.71	24.66
AMF2h	2	23.19	23.89	25.12	26.38	25.53	28.48	24.95	23.52	25.57	24.81
AMF2h	2	23.14	24.47	25.34	26.97	25.71	26.65	25.10	23.53	26.46	24.64
AMF2h	2	23.25	24.04	25.39	27.02	25.96	26.92	25.69	23.51	25.71	25.68
AMF2h	3	24.85	25.45	26.87	26.48	28.26	29.31	26.07	25.76	29.07	26.17
AMF2h	3	24.59	25.34	27.44	26.69	27.47	29.39	26.97	25.54	28.12	26.64
AMF2h	3	25.13	25.37	27.27	26.62	27.43	29.09	26.95	24.86	28.88	26.62
AMF+Salt2h	1	22.84	24.48	24.60	27.69	25.33	26.52	24.35	23.28	25.93	24.83
AMF+Salt2h	1	22.72	24.17	23.39	26.41	25.60	26.31	24.48	23.93	25.81	24.76
AMF+Salt2h	1	22.86	23.82	24.00	26.77	26.26	27.71	24.69	24.19	26.10	25.65
AMF+Salt2h	2	22.18	22.94	23.06	28.07	24.73	26.69	23.76	23.70	25.04	24.28
AMF+Salt2h	2	21.95	23.09	23.15	27.74	25.23	27.06	23.34	24.05	25.09	24.79
AMF+Salt2h	2	22.25	23.43	23.52	27.43	24.36	26.33	23.28	24.91	24.57	24.42
AMF+Salt2h	3	20.97	22.15	22.68	26.85	24.43	26.83	22.11	24.01	24.01	22.83
AMF+Salt2h	3	21.47	22.12	22.31	26.47	24.63	26.90	21.96	23.54	24.02	22.98
AMF+Salt2h	3	21.13	22.24	22.82	26.93	23.95	26.49	22.16	23.34	24.05	23.41
Ct11d	1	23.32	24.37	25.61	26.67	25.19	27.82	25.74	25.12	28.16	26.25
Ct11d	1	23.52	24.60	25.94	26.90	24.61	26.98	25.44	25.87	27.60	25.76

**Treatment**	BR/TR	RG 1 ***ARP3***	RG 2 ***SAC1***	RG 3 ***VFB1***	RG 4 ***ATKRS-1***	RG 5 ***TUBB2***	RG 6 ***GRIK2***	RG 7 ***MYB4***	RG 8 ***CpOXS2***	RG 9 ***RSH1***	RG 10 ***HAG2***
Ct11d	1	23.59	24.36	24.89	26.71	25.01	28.98	25.16	25.59	28.19	25.73
Ct11d	2	22.00	23.06	22.86	24.81	23.27	25.04	24.27	23.94	24.78	23.53
Ct11d	2	21.51	22.80	23.41	23.80	23.64	25.09	23.79	23.51	25.66	23.63
Ct11d	2	21.55	22.97	23.15	23.74	23.66	24.26	24.32	23.08	25.11	23.28
Ct11d	3	21.92	23.35	21.95	23.97	24.64	25.14	23.87	22.70	25.02	23.90
Ct11d	3	22.14	23.65	22.16	24.34	24.73	25.72	23.32	22.82	24.55	24.03
Ct11d	3	21.97	23.81	22.22	24.64	24.81	24.88	23.67	22.77	25.29	23.57
Salt11d	1	21.57	22.48	21.94	26.22	24.12	25.15	23.17	22.16	24.70	23.56
Salt11d	1	22.32	22.95	22.48	25.75	23.41	25.29	23.79	22.53	24.41	22.87
Salt11d	1	21.95	22.72	22.21	25.99	23.77	25.22	23.48	22.35	24.56	23.22
Salt11d	2	24.30	24.21	24.26	26.93	27.84	27.88	26.05	24.64	26.94	25.25
Salt11d	2	24.05	24.10	24.69	27.30	26.59	27.45	25.43	23.64	26.48	24.97
Salt11d	2	24.15	24.03	23.85	27.17	26.77	26.91	25.66	23.45	26.83	25.79
Salt11d	3	24.34	24.28	24.65	27.90	28.05	27.19	24.77	24.38	26.72	25.21
Salt11d	3	25.15	24.50	24.15	28.35	27.67	26.97	25.25	24.75	26.56	24.63
Salt11d	3	25.18	23.70	24.44	27.43	27.44	27.17	25.87	24.87	27.40	24.94
AMF11d	1	21.99	22.76	23.79	27.15	24.73	27.18	22.42	26.66	24.07	23.45
AMF11d	1	21.89	21.84	24.04	25.64	25.20	27.55	22.50	24.32	24.31	23.11
AMF11d	1	22.03	22.82	24.34	26.89	25.25	27.60	21.84	24.84	24.62	23.17
AMF11d	2	21.63	22.43	22.24	27.48	23.63	27.25	22.97	24.16	25.23	23.40
AMF11d	2	21.97	22.19	22.88	26.62	23.74	26.85	22.53	23.79	25.13	23.28
AMF11d	2	21.58	22.05	23.64	26.94	23.89	26.01	22.21	23.64	25.18	23.44
AMF11d	3	21.38	22.06	21.85	25.22	23.20	23.99	22.29	21.94	24.32	22.58
AMF11d	3	21.77	21.85	21.74	25.01	22.84	24.33	22.92	21.99	24.68	22.81
AMF11d	3	21.80	21.55	21.38	25.33	23.33	23.77	22.75	21.80	24.43	22.90

**Treatment**	BR/TR	RG 1	RG 2	RG 3	RG 4	RG 5	RG 6	RG 7	RG 8	RG 9	RG 10
		ARP3	***SAC1***	***VFB1***	***ATKRS-1***	***TUBB2***	***GRIK2***	***MYB4***	***CpOXS2***	***RSH1***	***HAG2***
AMF+Salt 11d	1	21.89	22.97	24.04	27.49	25.46	27.80	23.16	24.39	24.84	23.79
AMF+Salt 11d	1	22.83	23.20	24.20	27.50	26.01	27.41	23.65	24.58	25.46	23.97
AMF+Salt 11d	1	22.59	22.99	23.95	28.39	25.50	27.40	23.52	23.48	25.27	23.44
AMF+Salt 11d	2	22.62	23.90	24.12	28.20	25.36	25.91	26.77	24.49	24.37	23.71
AMF+Salt 11d	2	22.19	23.10	24.33	27.88	26.08	27.41	26.91	23.44	25.15	23.98
AMF+Salt 11d	2	22.84	23.14	24.21	28.03	26.07	25.71	26.96	24.03	24.85	24.05
AMF+Salt 11d	3	22.94	23.20	24.56	26.10	26.14	25.49	23.01	23.09	25.27	24.15
AMF+Salt 11d	3	22.76	23.07	24.09	26.19	26.79	26.04	23.13	22.68	24.42	23.80
AMF+Salt 11d	3	22.85	23.14	24.33	26.15	26.47	25.77	23.07	23.79	25.30	24.29
Mean		22.86	23.53	23.90	26.52	25.83	26.37	24.26	23.58	25.78	24.41
NTC		-	-	-	-	-	-	-	-	-	-

Legend: RG (Reference Gene); NTC (no template control); BR (biological replicate); TR (technical replicate); *ARP3* (Actin-related protein 3); *SAC1* (Phosphoinositide phosphatase); *VFB1* (Vier F-box protein1); *ATKRS-1* (Lysyl-tRNA Synthetase); *TUBB2* (Tubulin beta-2 chain); *GRIK2* (Serine/threonine-protein kinase); *MYB4* (Transcription repressor MYB4); *CpOXS2* (Zinc finger CCCH domain-containing protein 30); *RSH1* (Putative GTP diphosphokinase); *HAG2* (Histone acetyltransferase type B catalytic subunit).
