# Supplementary material for: Reference genes for quantitative real-time PCR normalization of Cenostigma pyramidale roots under salt stress and mycorrhizal association
Source: Genet Mol Biol. 2021 May 31;44(2):e20200424. doi: 10.1590/1678-4685-GMB-2020-0424 (PMC8167929; doi:10.1590/1678-4685-GMB-2020-0424)
Supplement: Figure S1- [file 1415-4757-GMB-44-2-e20200424-s3.pdf]

Genetics and Molecular Biology
https://doi.org/10.1590/1678-4685-GMB-2020-0424

Supplementary Material to "Reference genes for quantitative real-time PCR normalization of *Cenostigma pyramidale* roots under salt stress and mycorrhizal association"

Figure S1 - Melting temperature of ten candidates reference genes (RGs) and two target transcripts (TTs).

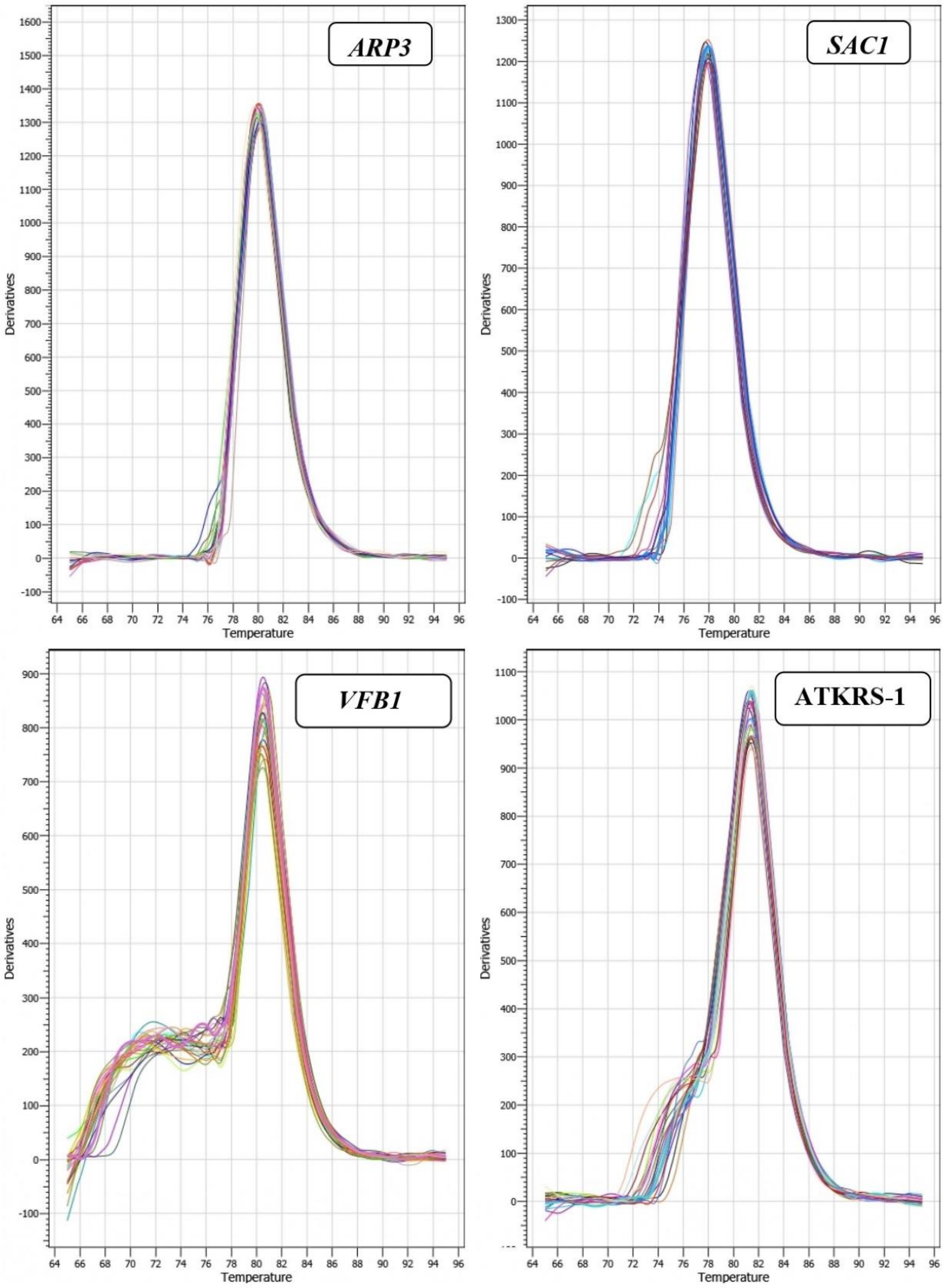

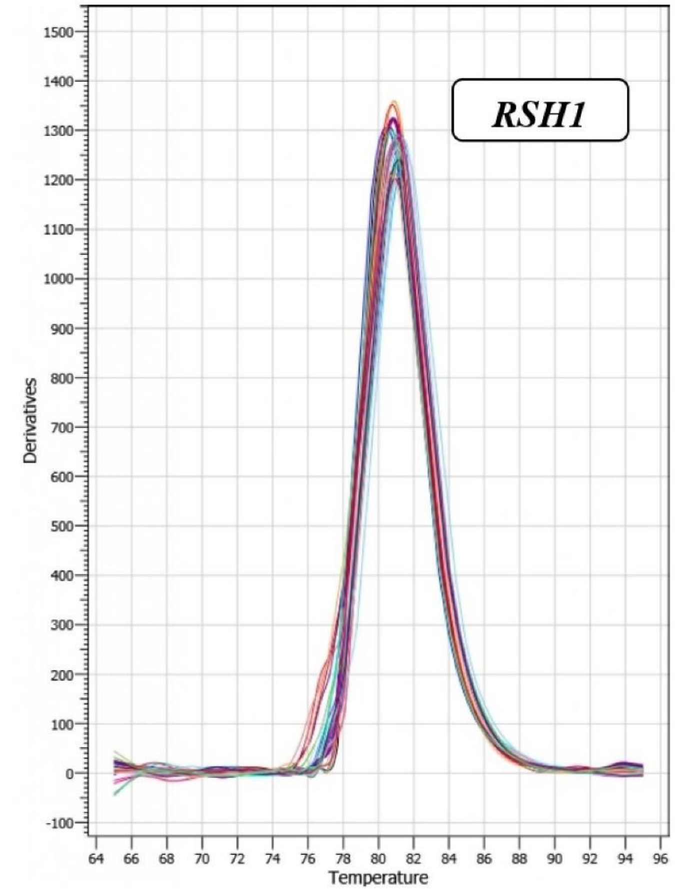
RSH1
Derivatives
Temperature
1500
1400
1300
1200
1100
1000
900
800
700
600
500
400
300
200
100
0
-100
64 66 68 70 72 74 76 78 80 82 84 86 88 90 92 94 96

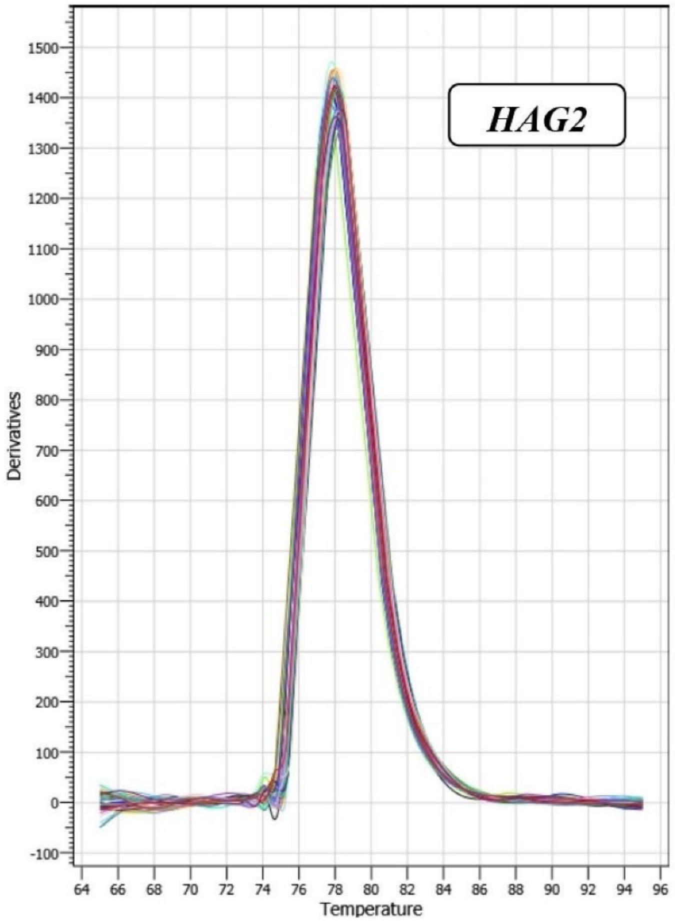
HAG2
Derivatives
Temperature
1500
1400
1300
1200
1100
1000
900
800
700
600
500
400
300
200
100
0
-100
64 66 68 70 72 74 76 78 80 82 84 86 88 90 92 94 96

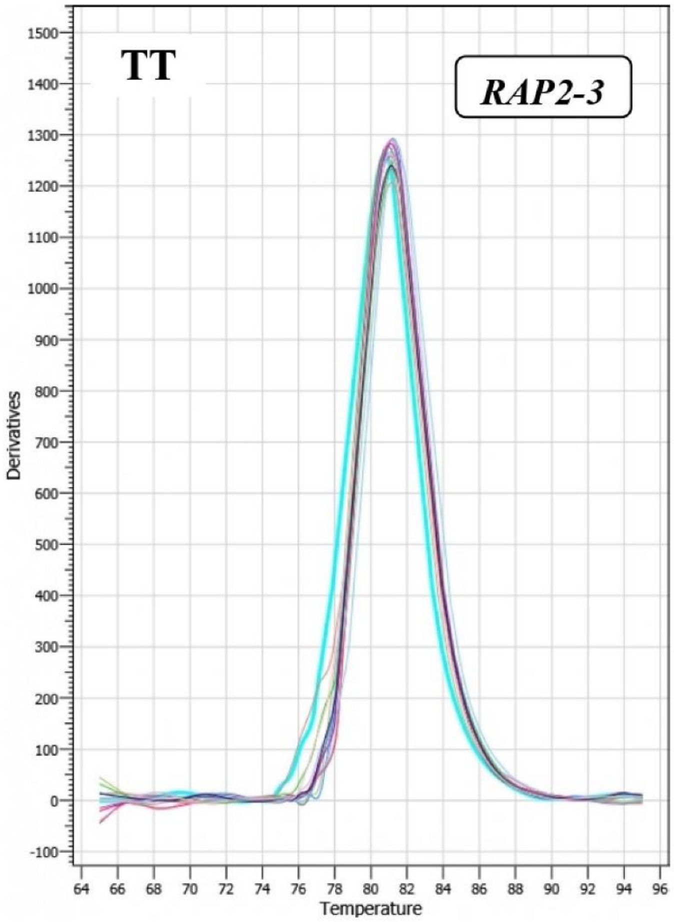
TT
RAP2-3
Derivatives
Temperature
1500
1400
1300
1200
1100
1000
900
800
700
600
500
400
300
200
100
0
-100
64 66 68 70 72 74 76 78 80 82 84 86 88 90 92 94 96

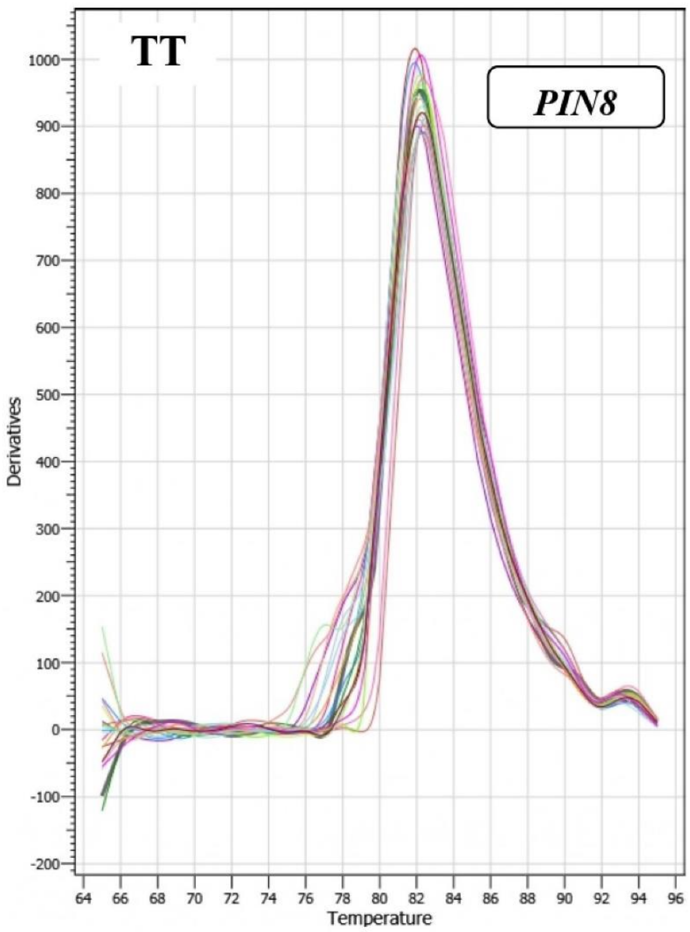
TT
PIN8
Derivatives
Temperature
1000
900
800
700
600
500
400
300
200
100
0
-100
-200
64 66 68 70 72 74 76 78 80 82 84 86 88 90 92 94 96

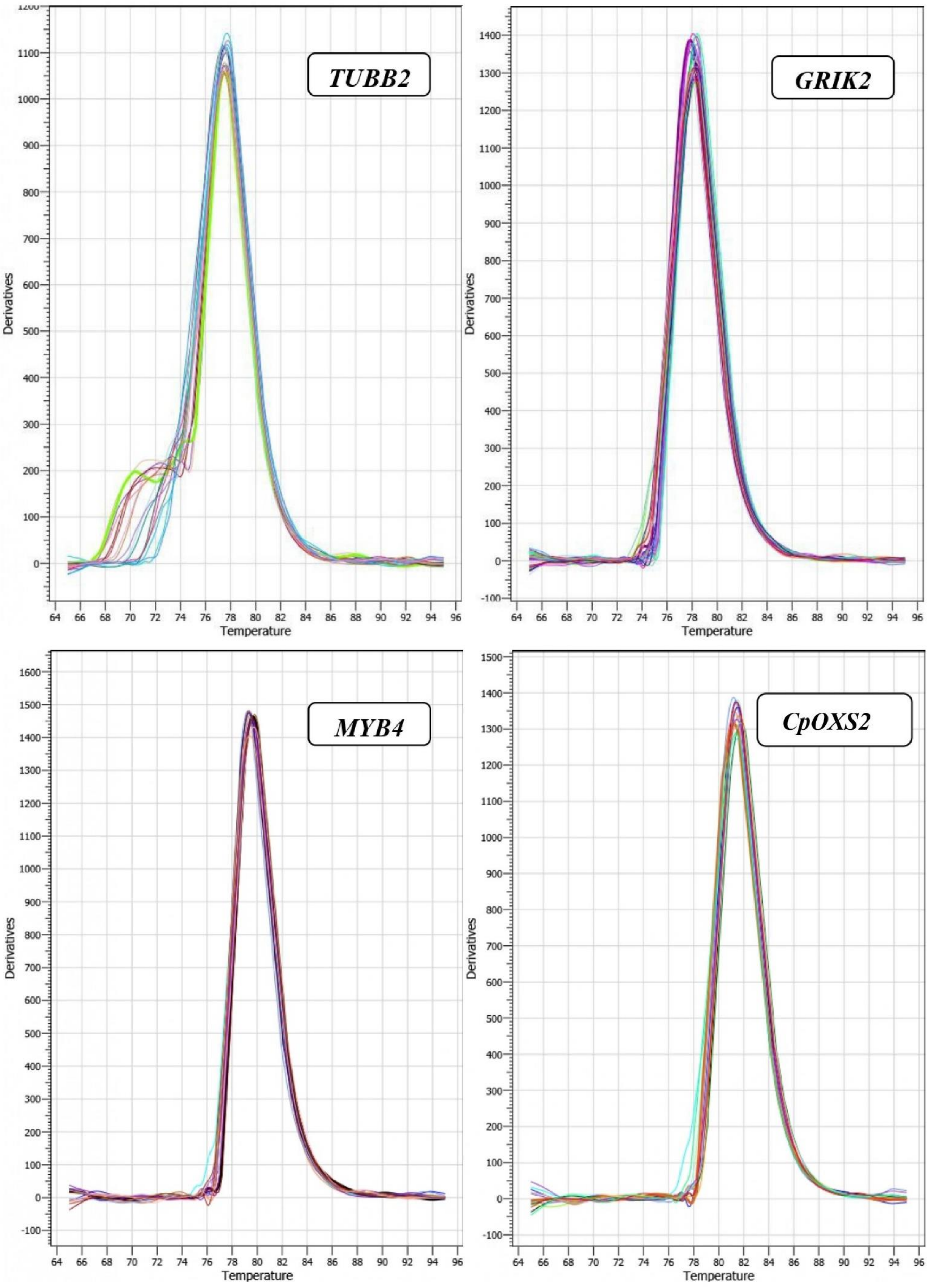
TUBB2
GRIK2
MYB4
CpOXS2
Derivatives
Temperature
